# Against Lung Cancer Cells: To Be, or Not to Be, That Is the Problem

**DOI:** 10.1155/2012/659365

**Published:** 2012-02-01

**Authors:** Naoko Okumura, Hitomi Yoshida, Yasuko Kitagishi, Yuri Nishimura, Shio Iseki, Satoru Matsuda

**Affiliations:** Department of Environmental Health, Nara Women's University, Kita-Uoya Nishimachi, Nara 630-8506, Japan

## Abstract

Tobacco smoke and radioactive radon gas impose a high risk for lung cancer. The radon-derived ionizing radiation and some components of cigarette smoke induce oxidative stress by generating reactive oxygen species (ROS). Respiratory lung cells are subject to the ROS that causes DNA breaks, which subsequently bring about DNA mutagenesis and are intimately linked with carcinogenesis. The damaged cells by oxidative stress are often destroyed through the active apoptotic pathway. However, the ROS also perform critical signaling functions in stress responses, cell survival, and cell proliferation. Some molecules enhance radiation-induced tumor cell killing via the reduction in DNA repair levels. Hence the DNA repair levels may be a novel therapeutic modality in overcoming drug resistance in lung cancer. Either survival or apoptosis, which is determined by the balance between DNA damage and DNA repair levels, may lender the major problems in cancer therapy. The purpose of this paper is to take a closer look at risk factor and at therapy modulation factor in lung cancer relevant to the ROS.

## 1. Introduction

Lung cancer is the commonest fatal cancer whose risk is dependent on the number of cigarettes smoked per day as well as the duration years of the smoking [1, 2]. Passive smoking also damages health [3]. Cigarette smoke is a complex mixture of more than 5000 chemicals that have been identified in the smoke. Among them, more than 50 are known to cause cancer in humans. A wide variety of the other toxic substances such as asbestos, polycyclic aromatic carbohydrates, arsenic, and diesel emissions also have been identified as potential causes of lung cancer [4, 5]. Some of these carcinogens react covalently with DNA to cause oxidative damage, which can induce DNA breaks [6]. Another recognized lung carcinogen is the chemically nearly inert gas radon [7], a ubiquitous natural air pollutant arising from radioactive decay of the uranium-238, which is present throughout the earth crust. Radon is a naturally occurring radioactive gas with the atomic number 86. It is odorless and colorless. Both radon-induced ionizing radiation and some components of cigarette smoke induce oxidative stress by transmitting or generating reactive oxygen species (ROS). The chronic exposure to ROS contributes to a variety of processes, including aging, degenerative diseases, and cancer [8]. ROS also appear to play an essential role as secondary messengers in the normal regulation of a variety of physiological processes, such as apoptosis, survival, and proliferative signaling pathways [9, 10]. As ROS are produced in all mammalian cells from mitochondrial oxidative respiration, cellular defense mechanisms have evolved to protect cells from ROS [11]. Those include DNA repair systems and detoxifying scavenger enzymes such as superoxide dismutases [12]. An imbalance between the mechanisms that generate and protect against ROS results in oxidative damage including the DNA damage, which results in DNA strand breaks. DNA damage and the DNA breaks threat to cells because it may cause mutations and alterations of chromosomal structures. These are intimately linked with cellular transformation [13]. Administration of NAC, a direct scavenger of ROS, prevents tumorigenesis in p53 null mice via suppression of ROS levels [14]. Loss of ROS level control may be critical for cellular phenotypes associated with cancer.

**Figure 1:**
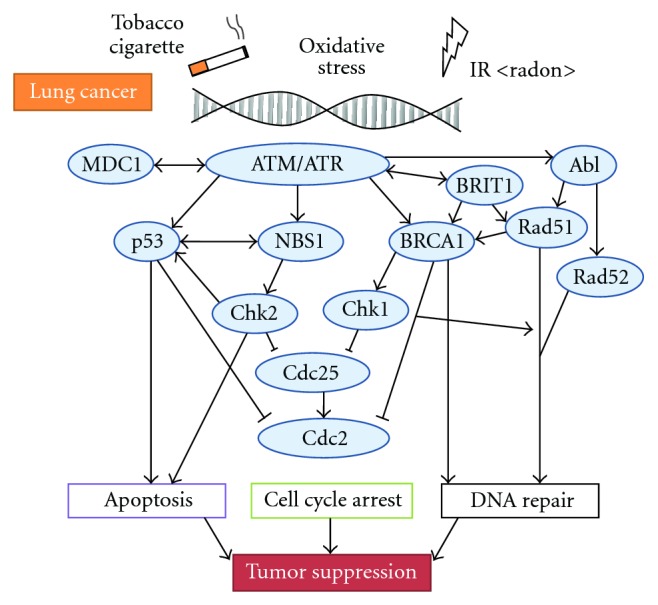
Schematic representation of the DNA repair, cell cycle arrest, and apoptosis signaling pathways. Examples of the molecule known to act on the regulatory pathways are shown.

Cells possess a machinery to maintain the genomic integrity in response to oxidative stresses. Under the genotoxic oxidative conditions, cells do not progress into S or M phase by activating DNA damage checkpoint [15]. The DNA damage checkpoint acts as a process to transmit information from damaged DNA lesions to cell cycle regulators, which permits cell a genomic adaptation to acquire a growth advantage. Mutations in several genes which mitigate the effects of DNA damage are known to predispose to develop a cancer. For example, mutations in ataxia telangiectasia-mutated (ATM) have been associated with increased risk of development of lung cancer [16]. ATM is a checkpoint kinase that phosphorylates a large number of proteins in response to radiation-induced DNA damage, including p53, Chk2, and BRCA1. Mouse knock-outs of the gene encoding an ROS scavenger or an antioxidant protein indicate susceptibility to tumors [17]. Smoking and radon exposure are surely major causes of lung cancer. The relative risk for lung cancer in current smokers is up to 20-fold higher than never smokers. However, only a fraction of cigarette smokers develop lung cancer suggesting individual differences in susceptibility. It has been hypothesized that these differences may be due to genetic variations in DNA repair machinery (Figure 1). In the present paper, we summarize the function of DNA repair molecules at a viewpoint of carcinogenic DNA damage and cancer therapy modulation involved in lung cancer. 

## 2. Smoking and Radon Involved in Lung Cancer

An estimated more than 80% of new cases of lung cancer are due to active cigarette smoking [18]. Although most patients with lung cancer are still men, the percentage of women has been rising steadily in recent years. The cigarette smoking is also a contributor to the development of a wide range of other malignancies such as oral, pharynx, esophagus, stomach, kidney, bladder, pancreas, and uterine cervix cancers as well as leukemias [19]. Nuclear DNA is the target of a number of different chemical structures present in cigarette. These genotoxins such as polycyclic aromatic hydrocarbons, in particular, benzo pyrene, and nitrosamines comprise the tobacco carcinogen biomarkers including the DNA adducts of benzopyrene, nitrosamines, alkylating agents, aldehydes, and the products of oxidative damage such as 8-oxo-dGuo [20]. Benzopyrene is a well-established carcinogen by forming DNA adducts and DNA double-strand breaks [21]. Accumulation of these genetic changes at multiple loci leads to progressive genomic damage and instability. The tumorigenic relevance of this DNA damage and instability is revealed by a series of studies, indicating that smokers with less efficient DNA repair capacities are at higher risk for developing lung cancer [22].

**Figure 2:**
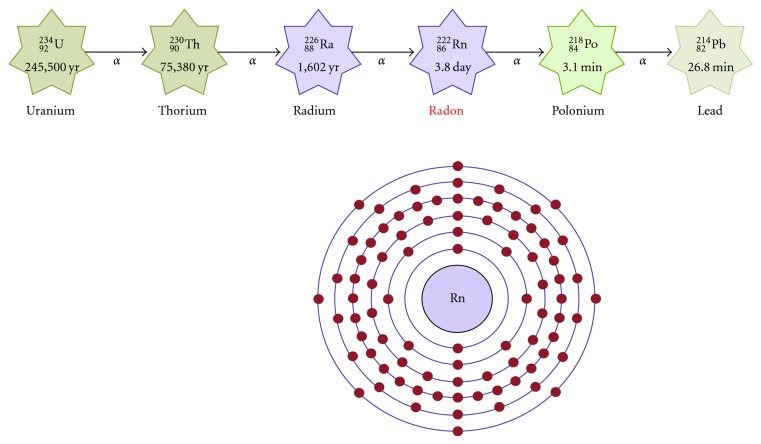
Schematic representation of decay chain of uranium series and an electron shell diagram for radon. Atomic number 86 radon (Rn) is a radioactive noble gas occurring as the decay product of uranium, thorium, or radium.

Epidemiological studies of uranium mine workers and experimental animal studies have suggested a positive correlation between exposure to alpha particles emitted from radon (^222^Rn: Figure 2) and the development of lung cancer [23]. It is well recognized that cellular responses to alpha particles include chromosome aberrations, genetic mutations, and induction of chromatid exchanges. As the effects of alpha particles may be related to the particle-associated production of ROS, radon can damage DNA indirectly via mechanisms involving cellular generation of the ROS. In most countries radon is the largest source of exposure to natural ionising radiation. Outdoor radon concentrations are usually low, but indoors they are higher in small buildings. The risk from indoor radon used to be estimated indirectly by extrapolation from risks seen in miners exposed to radon. Direct evidence has become available on the risk of lung cancer from indoor radon in people with cigarette smoking histories. Radon is the second most important cause of lung cancer in the general population. The risk of lung cancer due to the radon has shown that each 100 Bq/m^3^ of measured long-term radon exposure raises the relative risk by 16% in the estimation of exposure [24]. The relevance of other environmental risk factors such as asbestos or polycyclic aromatic carbohydrates to health is considered to be markedly lower than radon. The solid daughter of inhaled short-lived radon may deposit on the bronchial epithelium. Biological evidences have suggested that cells exposed to even a single alpha particle become damaged. The risk of cancer is dose dependent and proportional to the number of cells exposed to the alpha particle. Studies of radon-related lung cancer have quantified the risk in terms of radon concentration rather than radiation dose [25]. Consequently, policies to control radon are usually formulated in terms of radon concentration. The interaction of radiation and smoking exhibits a certain multiplicative relationship in the induction of lung cancer [26]. Smoking accounts for a large share of deaths in lung cancer attributed to radiation, which are higher in frequency than for the other solid cancers. A heavy smoker may accumulate an alpha radiation dose as high as 1 Gy to bronchial bifurcations. On the other hand, no increase in lung cancer among never smokers has been found in flight attendants exposed during flight to elevated background radiation [27]. Many countries already have policies to control cigarette smoking and the radon exposure.

## 3. DNA Repair Mechanism Involved in Cancer Development

**Figure 3:**
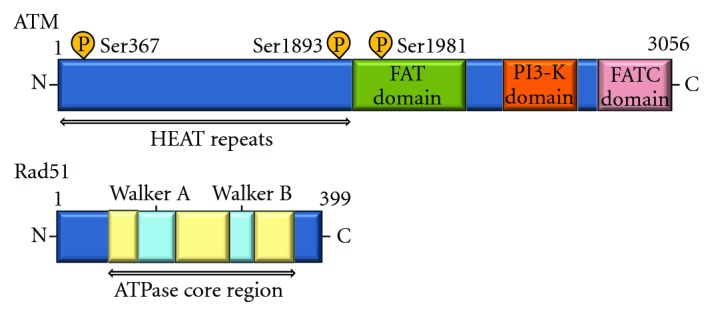
Schematic diagram indicating the domain structures of the ATM and Rad51 proteins. The autophosphorylation sites, HEAT (Huntington's elongation factor 3, a subunit of protein phosphatase 2A, TOR1) repeats, FAT (FRAP/ATM/TRRAP) domain, FATC (FAT C-terminal) domain in ATM, and Walker's box A and B domains (ATP binding) in Rad51 are also shown.

DNA adducts related to smoking are mainly repaired by the nucleotide excision repair pathway [28]. The pathway consists of about 30 proteins involved in DNA damage recognition, incision, and DNA ligation and synthesis. Repair of DNA double-strand breaks (DSBs) involves homologous and nonhomologous recombination repair pathways. These pathways include several molecules such as RAD51, ATM (Figure 3), ATM-, and Rad3-related (ATR), which are important for maintenance of genomic stability. DSBs create a major threat to genomic integrity of cells. Unrepaired or defectively repaired chromosomal irregularities may lead to cell apoptosis or tumorigenesis [29]. Lung cancer patients have been found to have lower DNA repair capacity compared with healthy individuals [30]. Studies have evaluated a small number of single nucleotide polymorphisms in a few DNA repair genes in lung cancer cells. In addition, molecular epidemiology studies have demonstrated that the variant DNA repair genotypes may alter susceptibility to lung cancer [31, 32].

ATM is essential for checkpoint and is one of key players in the initiation of DSBs repair. The ATM cDNA encodes 3056 amino acids protein of about 350 kDa protein [33]. The ATM protein, which is a kinase related to phosphoinositide 3 kinase, is activated by breaks in DNA or chromatin induced by oxidation stress. ATM protein phosphorylates many substrates such as BRCA1 and NBS1 at several serine residues after irradiation [34]. ATM activation in turn activates proteins such as p53, Chk2, BRCA1, and NBS1 that inhibit and/or modulate the cell cycle. ATM also controls the thiol-dependent histone acetylase-deacetylase system and may be involved in oxidative defense. It has been shown that impaired ATM function leads to defects in control of ROS. ATR is also well known as a member of phosphoinositide-3-kinase-related protein kinases and responds to single-strand DNA with ATR-interacting protein [35]. The ATR is involved in the phosphorylation of many proteins related to cell cycle checkpoints and DSBs repair pathways. More than 900 phosphorylation sites containing a consensus ATM and ATR phosphorylation motif (S/T-Q) in 700 proteins were identified by proteomic analysis. A DNA damage triggers ATM- or ATR-dependent pathways to control cell cycle progression, apoptosis, and DNA repair. However, how ATM and ATR are activated is not fully understood. As caffeine is an inhibitor of several cellular processes including activation of ATM and ATR, oral intake of caffeine in the drinking water of chronically irradiated mice suppressed UV-induced skin cancer development [36]. One of the downstream targets of ATM is nonreceptor tyrosine kinase Abl, which is phosphorylated and activated by ATM. The Abl and the interaction molecules [37, 38] are thought to relay apoptotic signals from ATM to p53.

After DSBs formation, the ATM-mediated DNA damage checkpoint pathway is activated by autophosphorylation and activation of ATM, which in turn phosphorylates Chk2 that initiates the phosphorylation of BRCA1 [39]. The BRCA1 germline mutations have been attributed to an increase in the risk of developing breast and ovarian cancer [40]. BRCA1 is rarely mutated in sporadic cancer cells, but epigenetic inactivation of BRCA1 has been documented. The gene encodes a multifunctional protein that has been implicated in regulation of the cell cycle, various transcriptional pathways, DNA damage signaling and repair, and sensitivity to chemo- and radiotherapy. Previous studies have shown that the BRCA1 stimulates antioxidant gene expression and protects cells against oxidative stress [41]. Wild-type BRCA1 but not a cancer-associated mutant significantly reduced the ROS levels. BRCA1 also reduced the levels of protein nitration and H_2_O_2_-induced 8-oxo-dGuo lesions in both carcinoma and nontumor cell lines DNA. Since peroxy-nitrite is formed by the reaction of superoxide with nitric oxide, the reduction may be due to a BRCA1-mediated reduction of superoxide [42].

Rad51 also participates in the repair of DSBs [43], which may cause genomic instability and cancer, by homologous recombination involving chromatids formed after the S phase. The Rad51 is the major strand-transfer protein in eukaryotic cells. Rad51 has been found to interact with many proteins including Ab1 protein kinase [44]. Rad51 had been shown to have ATPase activity *in vitro* and this ATPase activity is necessary for the recombination repair. A dominant-negative chimeric Rad51 protein suppresses the recombination. The level of the Rad51 protein is elevated in some tumor cell lines. A cell line expressing the oncogenic tyrosine kinase Abl had an enhanced level of Rad51 [44]. And p53 pathway also act to keep Rad51 expression. The elevated Rad51 resulted from both enhanced STAT5-dependent transcription and inhibition of caspase3-dependent cleavage. Rad51 has been implicated as a determinant of cellular radiosensitivity. When cells are exposed to genotoxic agents including irradiation, Rad51 protein is recruited to the sites of DNA damage and associated with nuclear matrix where it mediates the search for a homologous sequence during homologous recombination.

The MRE-RAD50-NBS1 (MRN) complex also plays a critical role in the DSBs repair pathway [45]. They function both in the nonhomologous end joining pathway as a sensor for DNA damage and in the homologous recombination pathway. In addition, they play a role in intra-S phase cell cycle checkpoint. Mice heterozygous for NBS1 mutation develop lung tumors [46]. In humans, rare mutations in NBS1 cause Nijmegen breakage syndrome, a disorder resulting in microcephaly, immunodeficiency, chromosome instability, and increased risk of cancer. As genetic variations in NBS1 could influence cancer development, increased expression of NBS1 has been found in smoking-related lung cancer.

## 4. Therapy Efficacy Related to DNA Repair Mechanism

Platinum-based drugs such as cisplatin that induce DNA damage are commonly used chemotherapy agents against a variety of cancers. The cisplatin cytotoxicity results from the formation of DNA adducts, which promote the development of DSBs during replication. Development of resistance to cisplatin is considered a major factor in disease relapse. Sensitization of non-small-cell lung cancer (NSCLC) cells to cisplatin is accomplished through the regulation of key components in the DNA-damage checkpoint pathway. For example, an enhanced DNA repair by the NBS1 complex is critical in driving the chemoresistance. The ATM activated by the cisplatin is phosphorylated during apoptosis. This results in higher Chk1 and Chk2 kinase activity. Activated Chk1 and Chk2 increase the expression of cell cycle checkpoint proteins, including Cdc25A and Cdc25C, leading to higher levels of G2/M arrest in tumor cells. Controlling these pathways may overcome cisplatin resistance and enhance therapeutic efficacy [47].

BRCA1 plays an important but complex role in the cell's response to chemotherapy. Several lines of evidence have indicated that the status of BRCA1 protein influences the ability of cells to respond to agents that cause DNA damage. Low BRCA1 levels correlate with increased sensitivity to DNA damaging agents such as cisplatin. Cells lacking BRCA1 are more sensitive to it. Overexpression of BRCA1 mRNA was strongly associated with poor survival in the chemotherapy-naive NSCLC patients. Conversely, a series of experiments have suggested that low levels of BRCA1 correlate with resistance to taxanes and vinca alkaloids. No BRCA1 is more resistant to taxanes. Overexpression of BRCA1 confers sensitivity to docetaxel and paclitaxel [48]. Analysis of mRNA expression levels in metastatic malignant effusions from NSCLC patients also revealed BRCA1 expression level as positively correlated to docetaxel sensitivity. Accordingly, chemotherapy customized according to BRCA1 expression levels is associated with excellent survival for NSCLC patients [49]. And BRCA1 may represent an ideal biomarker with the ability to predict response to a wide array of agents currently used in lung cancer therapy.

Abnormal expression of Rad51 has been reported in various malignant tumors. In general, Rad51 is expressed at higher levels in tumor cells as compared with normal cells [50]. Rad51 expression is increased in p53-negative cells, and since p53 is often mutated in tumor cells, there is a tendency for Rad51 to be overexpressed in tumor cells. It is the oncogenic activation of the Abl tyrosine kinase that is also responsible for the elevated Rad51 level [51]. The induction appears to be cell-type dependent. It is generally considered that radiation has no effect on Rad51 expression in mammalian cells. However, the induced expression of Rad51 within a tumor cell may reduce the cell's sensitivity to subsequent irradiations. Rad51 may be an appropriate target for selectively enhancing the radiosensitivity. As cells with increased Rad51 levels are more resistant to DNA damage, there is a selection for tumor cells to have higher Rad51 levels. In NSCLC, high-level Rad51 expression in cells generally confers resistance to ionizing radiation and resistance to chemotherapeutic agents. That may indicate poor prognostic outcome. Rad51 can protect lung cancer cells from cytotoxic effects induced by gefitinib. Suppression of Rad51 expression by small interfering RNA (si-Rad51 RNA) transfection can augment the cytotoxic effect of gefitinib, suggesting that Rad51 may be a novel lung cancer therapeutic modality to overcome drug resistance to gefitinib [52]. Erlotinib (Tarceva) is a selective epidermal growth factor receptor tyrosine kinase inhibitor used in the treatment of NSCLC. Knocking-down endogenous Rad51 expression by the si-Rad51 RNA significantly enhanced erlotinib-induced cytotoxicity. In contrast, overexpression of Rad51 by transfection with Rad51 vector could protect the cells from cytotoxic effects induced by the erlotinib. Phosphoinositide 3 kinase inhibitor (Wortmannin) suppressed the expression of Rad51 and enhanced the erlotinib-induced cell death in erlotinib-resistant cells [53]. The erlotinib attenuate radiation-induced Rad51 expression and enhance the radiation-induced apoptosis in NSCLC cells. Imatinib (Gleevec) is a relatively specific inhibitor of Abl tyrosine kinase. As the Abl can play a role in the regulation Rad51 expression, the imatinib treatment reduced Rad51 expression. And the pretreatment of the malignant cells with imatinib resulted in an enhancement in their radiosensitivity [54]. Because the generation of Rad51 after irradiation is assumed to contribute to the repair of DSBs, the reduction is likely responsible for the enhanced radiosensitivity. In addition, depletion of endogenous Rad51 expression by the si-Rad51 RNA significantly enhanced mitomycin C-induced cell death and cell growth inhibition. In contrast, overexpression of Rad51 protects lung cancer cells from the synergistic cytotoxic effects induced by mitomycin C and emodin (1,3,8-trihydroxy-6-methyl-anthraquinone). Emodin is a tyrosine kinase inhibitor and has anticancer effects on lung cancer. Rad51 is also involved in the sensitivity of small cell lung cancer to etoposide [55]. Thus, suppression of Rad51 expression may be considered as potential therapeutic modalities for lung cancer [56].

Histone deacetylase inhibitors (HDACis) represent a promising compounds for the treatment of lung cancer. Chk1 downregulation occurred after HDACi treatment preceding apoptosis. Ectopic expression of Chk1 overcomes HDACi-induced cell death. Inhibition of Chk1 showed strong synergistic effect with LBH589, a HDACi, in lung cancer cells, suggesting that Chk1 could be a marker to assess HDACi efficacy. Cells treated with AZD6244, an inhibitor of MEK1/2, had decreased Chk1 phosphorylation. And studies have indicated that the AZD6244 can enhance tumor cell radiosensitivity, suggesting that the effect involves an increase in mitotic catastrophe [57].

## 5. Perspectives

There is a growing interest in the chemotherapy-preventative ability of antioxidants in many products from vitamin C to resveratrol. These agents have been shown to lower ROS and the oxidative damage. The signaling function of ROS in the modulation of apoptotic and proliferative signaling pathways has suggested that ROS may be an important target for cancer chemoprevention. Then, lower incidences of cancer in people who consume a diet high in antioxidants might suggest that antioxidants could be used in cancer chemoprevention. A number of studies have examined the chemopreventative effect of the antioxidants; however, these studies have not provided consistent evidence in favor of such effects. Although there have been a lot of advances in understanding the molecular basis of tumorigenesis, lung cancer is still the nearly leading cause of death. There is a need to develop a methodology that can rapidly assess the potential carcinogenic properties of genotoxic agents present in atmosphere.

Molecular studies have demonstrated that the variant DNA repair genotypes may alter susceptibility to lung cancer. Actually, lung cancer patients have been found to have lower DNA repair capacity compared with healthy individuals at the initial stage. However, cancer cells can develop resistance through enhanced DNA damage repair, as DNA DSBs are the critical lesion in radiation-induced cancer cell death. The Rad51 plays an essential role in the repair for the DNA damage, and increased Rad51 can result in increased drug resistance. Rad51 has been reported to influence the outcome of patients treated with chemo-radiotherapy. In lung cancer, high expression of Rad51 in tumor tissue is associated with an unfavorable prognosis. Rad51 is therefore not only involved in the progression of carcinogenesis but also affects therapy resistance. The correlation between Rad51 levels and resistance to therapeutic agents suggests that targeted inhibition of Rad51 through the strategies using with a technology such as siRNA may improve the response to the treatments. Targeting other DNA repair molecules may also be an effective strategy for selectively enhancing tumor cell chemo-radiosensitivity. However, excessively reduced Rad51 levels can also result in genomic instability and rearrangements.

**Figure 4:**
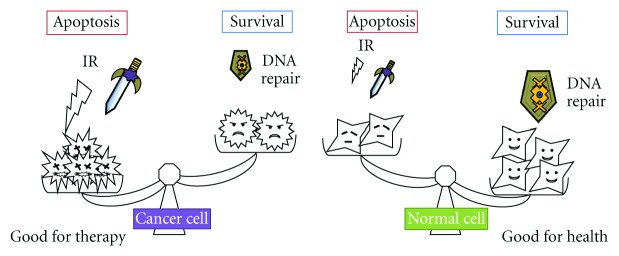
Survival or apoptosis, that is, the problem in cancer therapy. The determination either survival or apoptosis is due to the balance between DNA damage via IR or chemical agents and the DNA repair levels in cells.

It is clear that elimination of cancer cells by apoptosis is good for cancer therapy. In other words, survival of cancer cells is not good for the host health. Transgenic mice expressing the apoptosis-inhibitor survivin showed accelerated development of UV-induced squamous cell carcinomas [58]. On the other hand, therapy-associated lung injury results from cellular dysfunction generating the apoptotic cell-death mechanism. For example, the apoptotic mechanisms can explain the pathogenesis of radiation-induced pneumonitis. That is a so-called side effect associated with cancer therapy. To control cells for survival or apoptosis either in cancer cells or in normal cells is the most important problem in the therapy (Figure 4). Having too much radiation, ROS, and DNA repair levels may be as a double-edged sword. In recent years, a substantial research effort has aimed at developing new anticancer therapies with maximal effects and minimal adverse effects. Further studies on lung cancers will be necessary to determine the association of the DNA damage and the function of DNA repair-molecules with the relevant markers.
